# HMGB1/Advanced Glycation End Products (RAGE) does not aggravate inflammation but promote endogenous neural stem cells differentiation in spinal cord injury

**DOI:** 10.1038/s41598-017-10611-8

**Published:** 2017-09-04

**Authors:** Hongyu Wang, Xifan Mei, Yang Cao, Chang Liu, Ziming Zhao, Zhanpeng Guo, Yunlong Bi, Zhaoliang Shen, Yajiang Yuan, Yue Guo, Cangwei Song, Liangjie Bai, Yansong Wang, Deshui Yu

**Affiliations:** 1 Department of Orthopedic, First Affiliated Hospital of Jinzhou Medical University, Jinzhou City, PR China; 2 Department of Endocrinology, First Affiliated Hospital of Jinzhou Medical University, Jinzhou City, PR China; 3 Department of Stomatology, Second Affiliated Hospital of Jinzhou Medical University, Jinzhou City, PR China; 4 Department of Orthopedics, Second Hospital of Jinzhou, Jinzhou City, PR China; 5 Department of Orthopedics, China Medical University, Shenyang City, PR China

## Abstract

Receptor for advanced glycation end products (RAGE) signaling is involved in a series of cell functions after spinal cord injury (SCI). Our study aimed to elucidate the effects of RAGE signaling on the neuronal recovery after SCI. *In vivo*, rats were subjected to SCI with or without anti-RAGE antibodies micro-injected into the lesion epicenter. We detected Nestin/RAGE, SOX-2/RAGE and Nestin/MAP-2 after SCI by Western blot or immunofluorescence (IF). We found that neural stem cells (NSCs) co-expressed with RAGE were significantly activated after SCI, while stem cell markers Nestin and SOX-2 were reduced by RAGE blockade. We found that RAGE inhibition reduced nestin-positive NSCs expressing MAP-2, a mature neuron marker. RAGE blockade does not improve neurobehavior Basso, Beattie and Bresnahan (BBB) scores; however, it damaged survival of ventral neurons via Nissl staining. Through *in vitro* study, we found that recombinant HMGB1 administration does not lead to increased cytokines of TNF-α and IL-1β, while anti-RAGE treatment reduced cytokines of TNF-α and IL-1β induced by LPS via ELISA. Meanwhile, HMGB1 increased MAP-2 expression, which was blocked after anti-RAGE treatment. Hence, HMGB1/RAGE does not exacerbate neuronal inflammation but plays a role in promoting NSCs differentiating into mature neurons in the pathological process of SCI.

## Introduction

Spinal cord injury patients usually lose their self-care ability, bring suffering to families and burden society. There exists an irreversible neuronal cell death after primary injury, and expansion secondary injury finally induces axon tracts to break off and causes motor or sensory dysfunction. Secondary injury is mainly caused by metabolism disturbance, massive inflammatory response and neurotoxins that enlarge neuronal cell death^1–3^. The secondary injury of a spinal cord is a process that we try to prevent using drugs; however, we could still not find effective ways to stop this physiopathology. The mechanisms underlying SCI are still unknown. Inflammation after central nervous system (CNS) injury plays both detrimental and beneficial roles in the regeneration of neuron injuries. The injured cellular components of neuronal cells induce immune cell infiltration and excessive release of pro-inflammatory cytokines through binding with their receptors advanced glycation end products (RAGE)^4^. RAGE is the main receptor for amphoterin/High mobility group box-1 (HMGB1), advanced glycation end-products (AGEs), calgranulins, and amyloid-beta peptides to guide many cell functions such as inflammation, apoptosis, or proliferation in tissue homeostasis and regeneration, especially in CNS^5–8^.

There are neural stem cells in the spinal cord of adults^9^ that promote neurogenesis in development, growing and aging processes^10–12^. NSCs facilitate neurogenesis in traumatic brain injury and ischemic CNS disorder^13–16^. Endogenous NSCs may exhibit de novo neurogenesis at the damaged site during injury, although their self-repair ability is limited^15, 16^. Nestin expression is regarded as a reliable NSC marker and can characterize NSCs in CNS^17–22^. Nestin-positive stem cells can transform into astrocytes or other glial cells and promote nestin-positive cells in SCI that predominantly show a rise in neurons^23–25^. NSCs facilitate neuronal cell proliferation, migration and neurogenesis after SCI, which may bring hope for regeneration of the injured spinal cord. HMGB1 released from astrocytes promotes proliferation of NSCs through activating RAGE^26^.

In our study, we demonstrated the effect of RAGE blockade on the neural stem cells after SCI; we found that RAGE blockade suppressed endogenous nestin-positive stem cell transformation into mature MAP-2-positive cells. HMGB1 does not increase the cytokines of pro-inflammatory factors, TNF-a and IL-1β, while RAGE blockade attenuated LPS-induced pro-inflammatory factors in primary spinal neuron culture. We found that RAGE blockade destroys neuronal survival at the ventral horn and does not benefit the neurorecovery of the injured spinal cord. HMGB1/RAGE may play a role in endogenous neural stem cell differentiation in the process of SCI.

## Results

### Endogenous neural stem cells co-expressed with RAGE were activated after SCI; however, RAGE blockade reduced nestin overexpression induced by SCI

**Figure 1:**
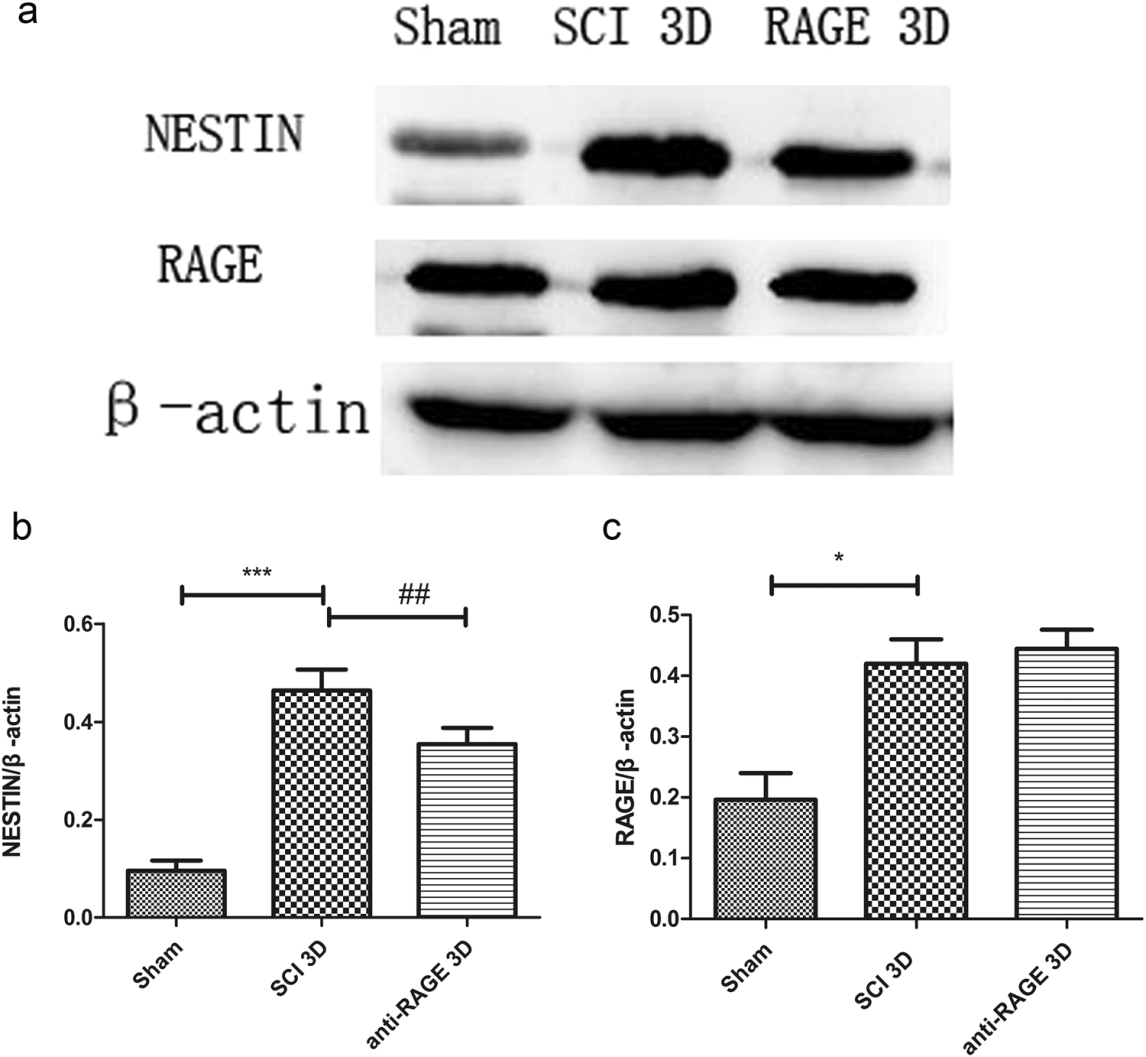
RAGE blockade further reduced nestin expression but showed no significant effect on RAGE expression 3 days after SCI. (**a**) The expressions of Nestin and RAGE via Western blot. (**b**) Quantitative analysis of Nestin expression in different groups. (**c**) Quantitative analysis of RAGE expression in different groups. Values are means ± SD. ^*^P < 0.05, ^***^P < 0.001 SCI group vs sham group, ^#^P < 0.05, ^##^p < 0.01anti-RAGE group vs SCI group.

Endogenous neural stem cells were activated and were able to transfer into the epicenter and differentiate into different phenotypes of neuronal cells after traumatic brain injury or ischemia^14–16^. RAGE was found widely expressed in the central nervous system, which associates with neuronal inflammation, neurite outgrowth and neuronal differentiation and plays a crucial role in the process of spinal cord injury^27^. We detected expression of nestin, a generally accepted neural stem cell marker, at 3 days after SCI with or without anti-RAGE antibody. Our results showed that nestin was activated at 3 days after SCI (SCI group vs sham group, 46.36 ± 5.28% and 9.59 ± 2.48%, p < 0.001). However, RAGE blockade reduced nestin expression after SCI (anti-RAGE group vs SCI group, 35.42 ± 4.09% and 46.36 ± 5.28%, p < 0.01; Fig. 1a,b). We observed increased RAGE expression at 3 days after SCI (SCI group vs sham group, 41.99 ± 4.92% and 19.62 ± 5.32% p < 0.05), while blockade of RAGE showed no significant effect on RAGE expression (anti-RAGE group vs SCI group, 44.49 ± 3.80%and 41.99 ± 4.92%; Fig. 1a,c).

**Figure 2:**
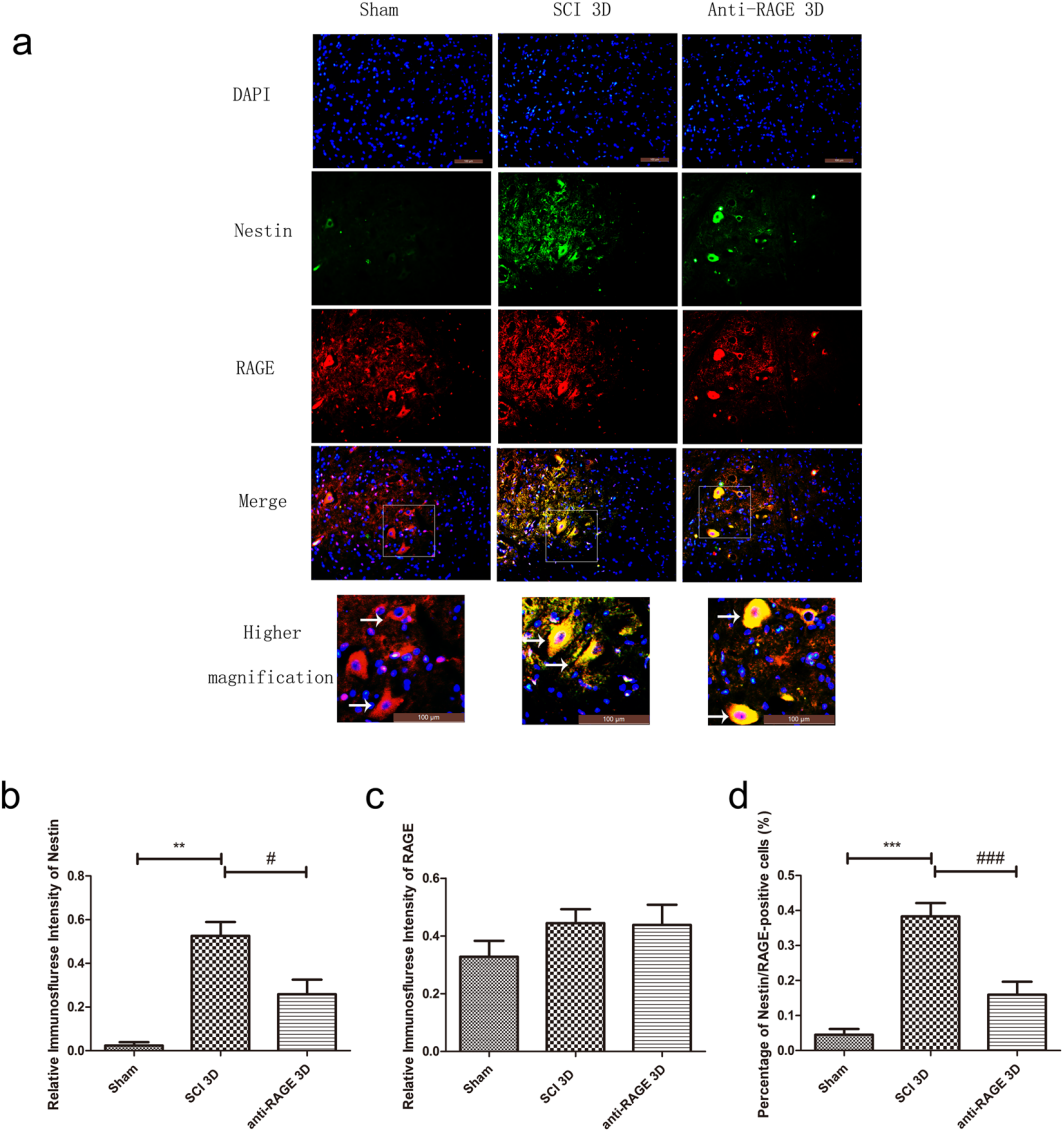
RAGE blockade reduced number of Nestin-positive NSCs that co-expressed RAGE 3 days after SCI. (**a**) Figures with the blue fluorescence dye are for the nucleus. Figures with the green fluorescence stand for nestin-positive cells. Figures with red fluorescence stand for RAGE-positive cells. Figures merged with DAPI/NESTIN/RAGE showed double-positive cells. In the sham group, nestin-positive cells can seldom be found, while RAGE expression can be found in neuronal cells. In the SCI group, we found increased nestin-positive and RAGE-positive cells expression after SCI. In the anti-RAGE group, we observed less nestin expression and most of nestin-positive cells were co-expressed with RAGE. Higher magnification shows the area of a white square, which represents magnification of *2.5, and the white arrow shows positive expression cells. Scar bar was 100 µm. (**b**) Relative immunofluorescence intensity of Nestin-positive cells. (**c**) Relative immunofluorescence intensity of RAGE-positive cells. (**d**) Percentage of Nestin/RAGE double-positive cells %.

To examine RAGE effect on nestin-positive neural stem cells, immunofluorescence(IF) labeling of DAPI/RAGE/Nestin was performed to confirm their expression in neural stem cells in SCI after RAGE blockade. Nestin expression was seldom found in the sham group, suggesting that neural stem cells were dormant in the spinal cord. However, at 3 days after injury, nestin-positive NSCs were activated near the spinal central canal (SCI group vs sham group, 15.26 ± 2.47% vs 0.18 ± 0.11%, *p* < 0.001; Fig. 2a–c) and co-expressed with RAGE. However, RAGE blockade partly suppressed nestin expression at 3 days after SCI. We observed activated RAGE expression at 3 days after SCI that was highly co-expressed with nestin. The percentage of Nestin/RAGE double-positive cells showed that increased nestin expression was partly inhibited by RAGE blockade (anti-RAGE group vs SCI group, 5.20 ± 0.87% and 15.26 ± 2.47%, *p* < 0.05; Fig. 2c).

**Figure 3:**
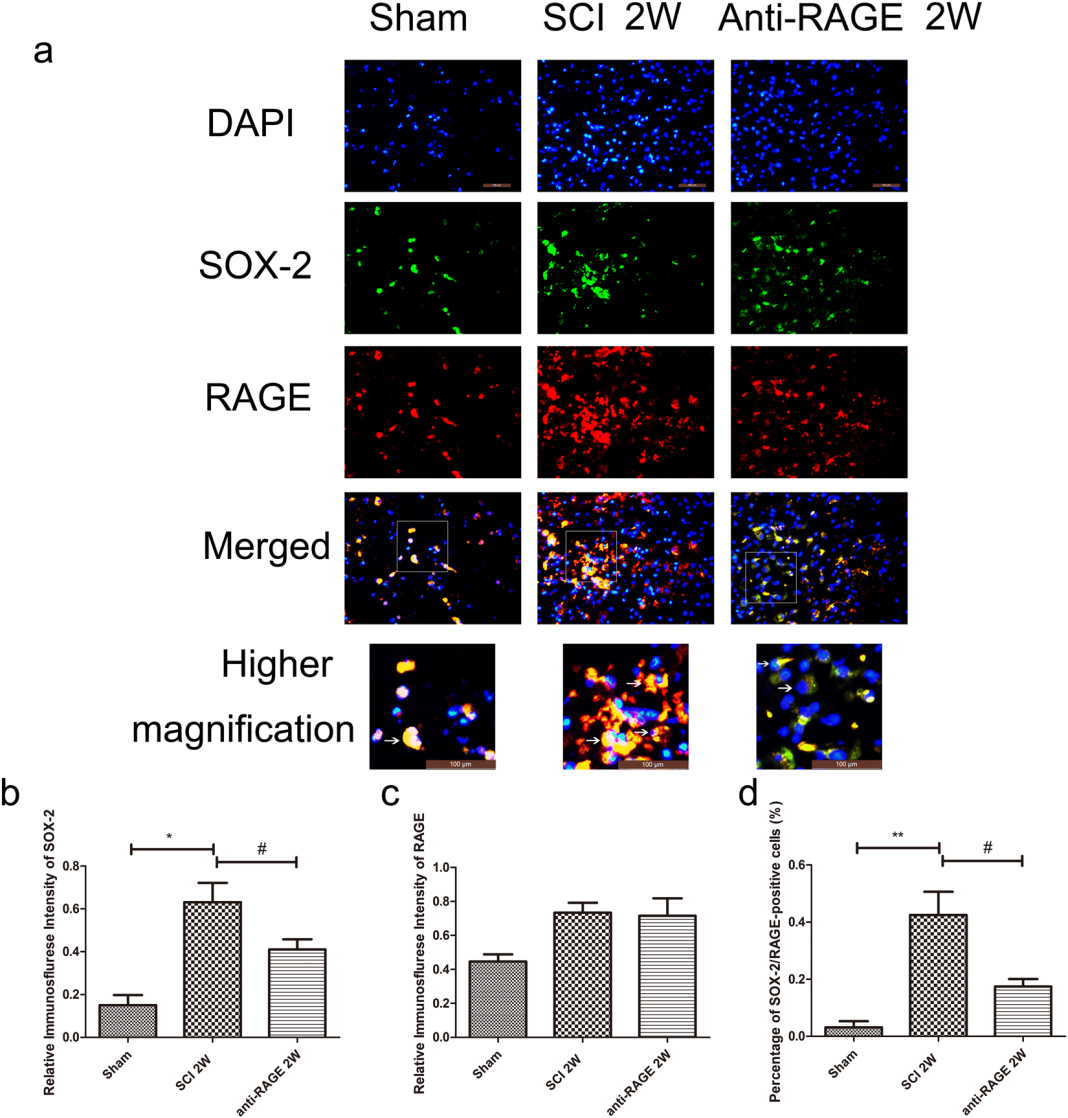
SOX-2 positive endogenous stem cells were activated 2 weeks after SCI, while RAGE blockade reduced SOX-2/RAGE double-positive cells. (**a**) Figures with the blue fluorescence dye are for the nucleus. Figures with the green fluorescence stand for SOX-2-positive cells. Figures with red fluorescence stand for RAGE-positive cells. Figures merged with DAPI/SOX-2/RAGE showed double-positive cells. In the sham group, SOX-2 positive cells were seldom found and rarely co-expressed with RAGE. At 2 weeks after SCI, increased SOX-2/RAGE double-positive cells were observed, but the anti-RAGE treatment group reduced SOX-2 expression at 2 weeks after injury. Higher magnification shows the area of a white square with magnification of *2.5, and the white arrow showed positive expression cells. Scar bar was 100 µm. (**b**) Relative immunofluorescence intensity of SOX-2-positive cells. (**c**) Relative immunofluorescence intensity of RAGE-positive cells. (**d**) Percentage of SOX-2/RAGE double-positive cell (%).

We further measured stem cell pluripotency marker SOX-2 at 2 weeks after SCI. RAGE/SOX-2 expression was investigated by IF labeling. In the sham group, few SOX-2-positive cells co-expressed with RAGE were found. 2 weeks after SCI, SOX-2 expression was activated (SCI group vs sham group, 42.45 ± 10.04% vs 3.13 ± 2.66%, *p* < 0.001; Fig. 3a,b). Relative expression of RAGE showed no significant differentiation (Fig. 3c). Reduced SOX-2/RAGE-positive cells can be found in the anti-RAGE treatment group at 2 weeks after injury(anti-RAGE group vs SCI group, 17.43 ± 3.16% and 42.45 ± 10.04%, *p* < 0.05; Fig. 3a,d), suggesting that RAGE may play a critical role in mediating endogenous neural stem cell function.

### RAGE blockade suppressed Nestin-positive neural stem cells transforming into MAP-2-positive mature neurons after SCI

**Figure 4:**
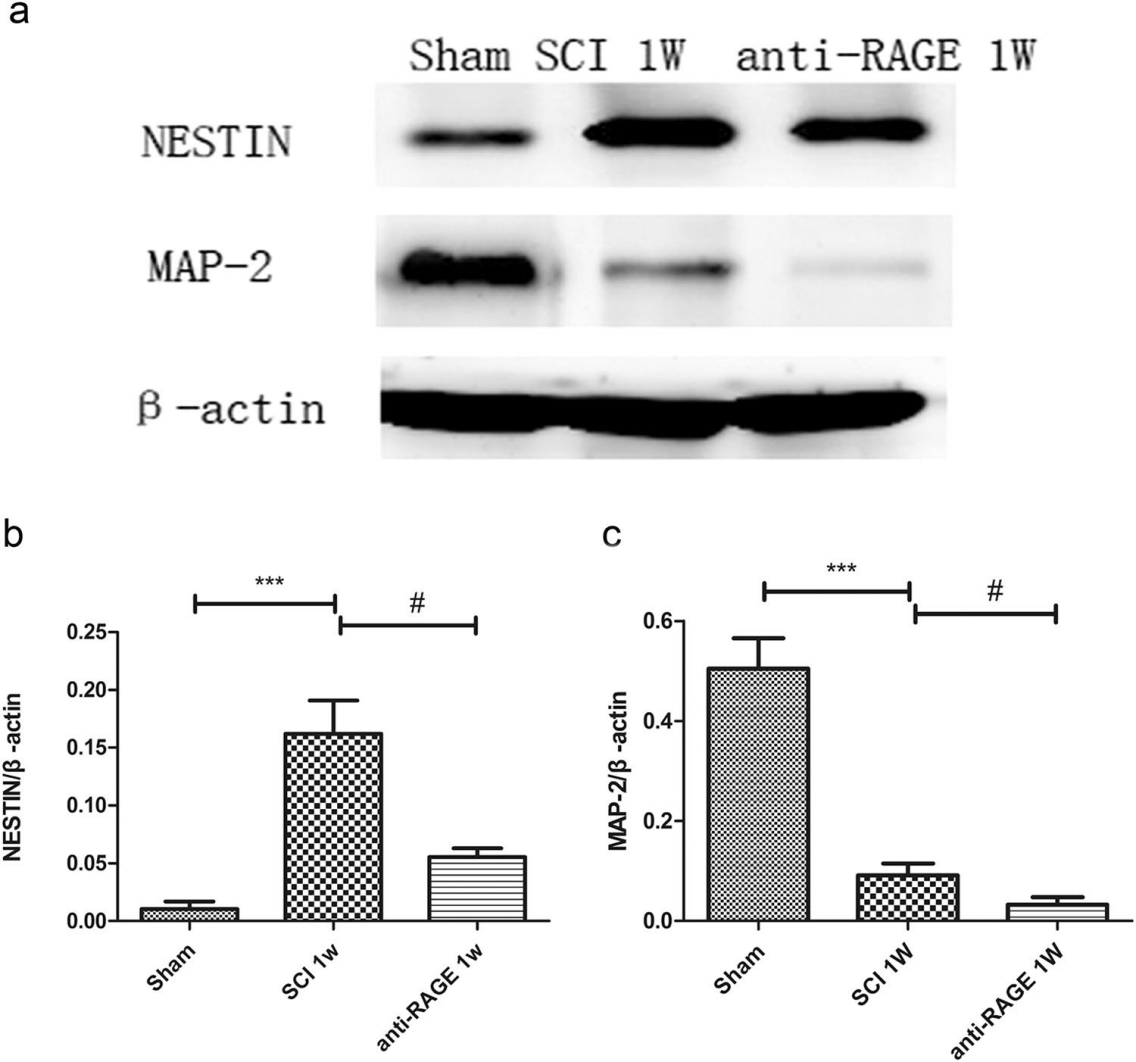
RAGE blockade reduced Nestin and MAP-2 expression 1 week after SCI via Western blot. (**a**) Western blot for nestin and MAP-2 expression. (**b**) Quantitative analysis of Nestin expression. (**c**) Quantitative analysis of MAP-2 expression.Values are means ± SD. *P < 0.05, ** P < 0.01, *** P < 0.001 SCI group vs sham group, ^#^P < 0.05,anti-RAGE group vs SCI group.

**Figure 5:**
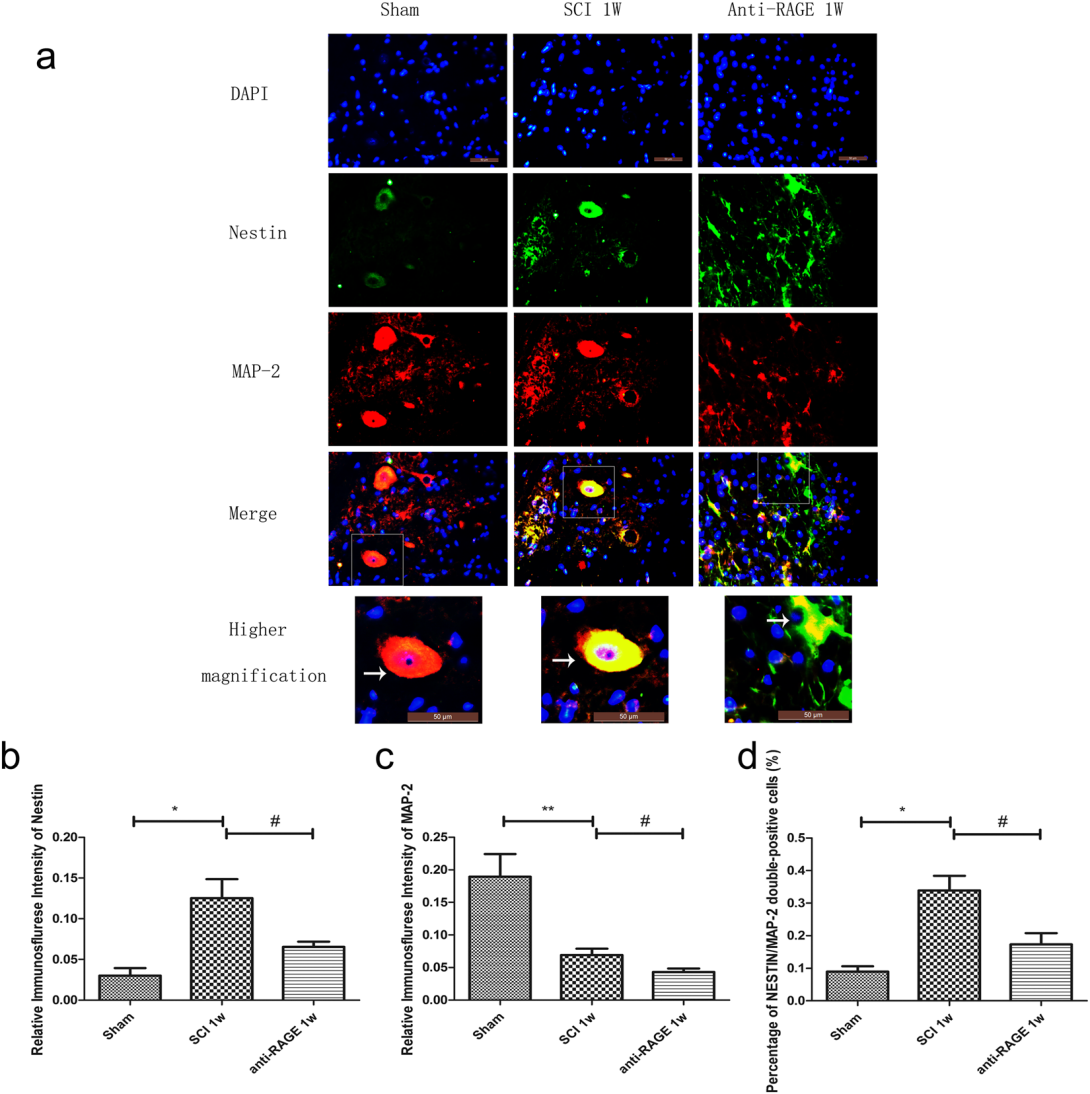
RAGE blockade reduced Nestin-positive cells expressing MAP-2 1 week after SCI. Figures with the blue fluorescence dye stand for the nucleus. Figures with the green fluorescence stand for nestin-positive cells. Figures with red fluorescence stand for MAP-2-positive cells. Figures merged with DAPI/NESTIN/RAGE showed double-positive cells. In the sham group, nestin expression was seldom found, but after SCI, nestin expression was significantly enhanced and co-expressed with MAP-2. In the anti-RAGE group, we observed the reduced nestin-positive cells were expressed with less MAP-2. Higher magnification shows the area of a white square with magnification of *1000. Scar bar was 50 µm. The white arrow shows positive expression cells. (**b**) Relative immunofluorescence intensity of SOX-2 expression. (**c**) Relative immunofluorescence intensity of RAGE expression. (**d**) Percentage of SOX-2/RAGE double-positive cell (%).

We assessed the expression of nestin till 1 week after SCI, and RAGE blockade suppressed nestin expression at 1 week after SCI, as measured via Western blots (SCI group vs sham group, 16.20 ± 3.52% and 1.04 ± 0.81, p < 0.001; Fig. 4a,b). We further detected expression of MAP-2, a mature neuron marker; MAP-2 expression was high in the sham group, but it was seriously reduced at 1 week after SCI (SCI group vs sham group, 9.15 ± 2.83% vs 50.44 ± 7.46%, p < 0.001; Fig. 4c). However, RAGE blockade treatment further decreased MAP-2 expression at 1 week after SCI(anti-RAGE group vs SCI group, 2.29 ± 1.78% vs 9.15 ± 2.83%, p < 0.05; Fig. 4a,c), indicating that RAGE signaling may promote the number of MAP-2-positive mature neurons. Via immunofluorescence, we observed that nestin-positive NSCs were activated at 1week after SCI, while nestin expression can be suppressed by RAGE blockade (anti-RAGE group vs SCI group, 6.54 ± 0.81% vs 12.52 ± 2.87%, p < 0.05; Fig. 5a,b). Nestin-positive endogenous neural stem cells were co-expressed with MAP-2 at 1 week after SCI. In the sham group, there existed MAP-2-positive and nestin-negative cells that may be the mature neurons. However, after SCI, nestin expression was significantly enhanced and co-expressed with MAP-2, indicating that nestin-positive NSCs were transforming into mature neurons. In the anti-RAGE group, nestin-positive cells expressed with less MAP-2 expression (Fig. 5a,c), suggesting a reduced neural stem cells differentiation. However, we analyzed the percentage of Nestin/MAP-2 double-positive cells, which showed that up-regulated double-positive cells were partly suppressed by RAGE blockade (anti-RAGE group vs SCI group, 17.31 ± 4.25% vs 33.88 ± 5.52%, p < 0.05; Fig. 5a,d), suggesting that RAGE may be involved in regulating neuronal stem cell differentiation into mature neurons.

### Recombinant HMGB1 administration does not increase cytokines of the inflammatory factors TNF-a and IL-1β, while RAGE blockade reduced LPS induction of the contents of cytokines

**Figure 6:**
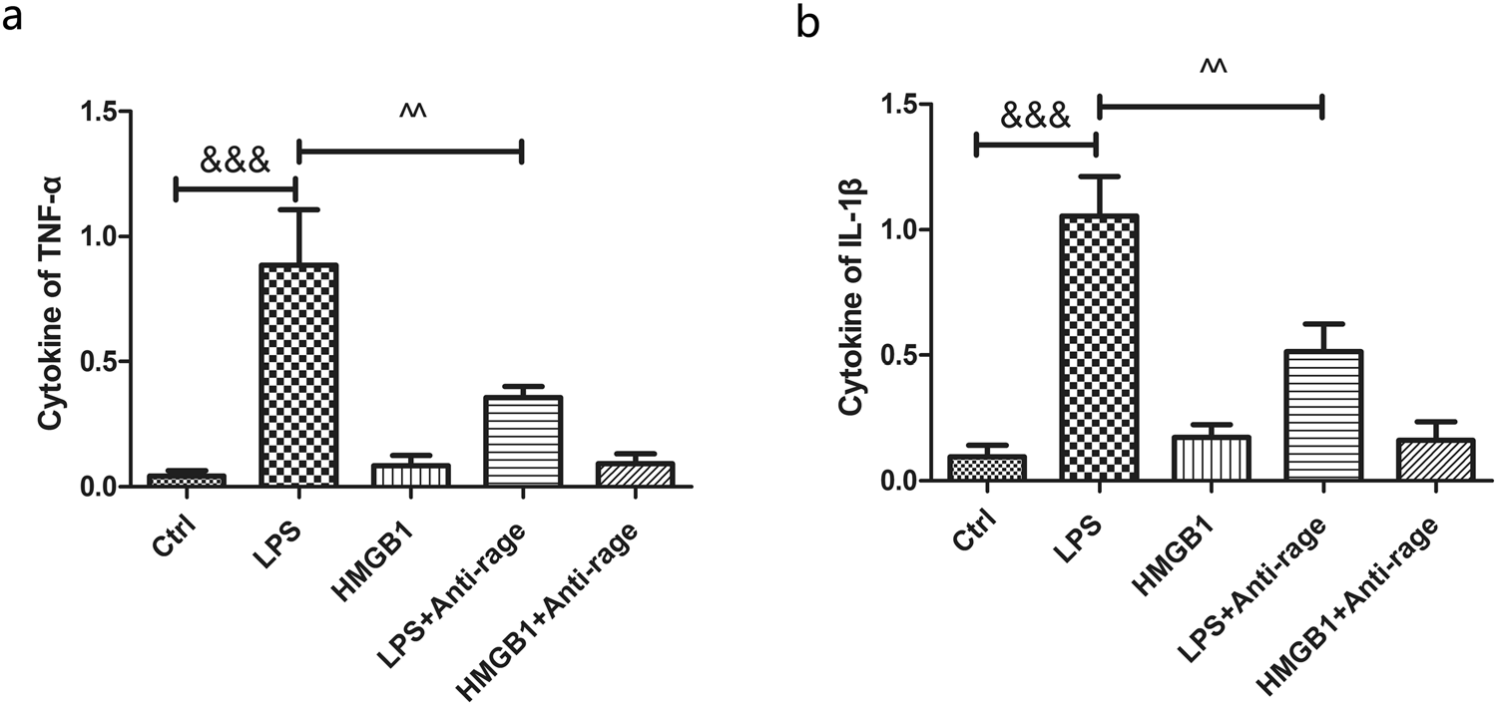
HMGB1 does not increase cytokines of TNF-α and IL-1β, but RAGE blockade reduced cytokines aroused by LPS in primary spinal neuron culture via ELISA. (**a**) Quantitative analysis of cytokines of TNF-α at 12 hours in the following groups: ctrl; LPS; HMGB1; LPS + anti-rage; and HMGB1 + anti-rage; (**b**) Quantitative analysis of cytokines of IL-1β in different groups. Values are means ± SD. ^&&^P < 0.01, ^&&&^P < 0.001 HMGB1 group vs ctrl group, ^^P < 0.01, ^^^p < 0.001LPS + anti-RAGE group vs LPS group.

HMGB1 released by both macrophages and neurons may modulate both inflammatory and axonal outgrowth in spinal cord injury^28^ and peripheral nerve regeneration^29^. RAGE and its ligand HMGB1 also promote neurite outgrowth and cell migration after injury^30–33^, but the effect can be blocked by anti-RAGE IgG^33^. Recombinant HMGB1 protein or anti-RAGE antibody was administered after being pretreated with LPS for 12 hours; after a 12-hour incubation, cytokines of TNF-a and IL-1β in the medium of the primary spinal neuron were examined via ELISA, and we found that HMGB1 administration does not increase cytokines of TNF-a and IL-1β, with no significant differentiation (HMBG1 group vs ctrl group, 8.39 ± 5.11% vs 4.34 ± 2.56% and 17.28 ± 6.35% vs 9.72 ± 5.44) after 12 hours, while LPS does increased cytokines of TNF-a and IL-1β (ctrl group vs LPS group; 4.34 ± 2.56 vs 88.56 ± 27.16% and 9.72 ± 5.44 vs 105.40 ± 19.26%, *p* < 0.001, Fig. 6a,b). However, RAGE blockade inhibited the cytokines of TNF-a and IL-1β induced by LPS (LPS + anti-RAGE group vs LPS group; 35.65 ± 5.48 vs 88.56 ± 27.16% and 51.38 ± 13.33 vs 105.40 ± 19.26%, *p* < 0.01, Fig. 6a,b), suggesting that HMGB1/RAGE does not directly increased pro-inflammatory factors, RAGE effect on cytokines may correlated with pathogen associated response.

### HMGB1 promotes MAP-2 expression, but it was partly abolished after treatment with anti-RAGE antibody in primary spinal neuron

**Figure 7:**
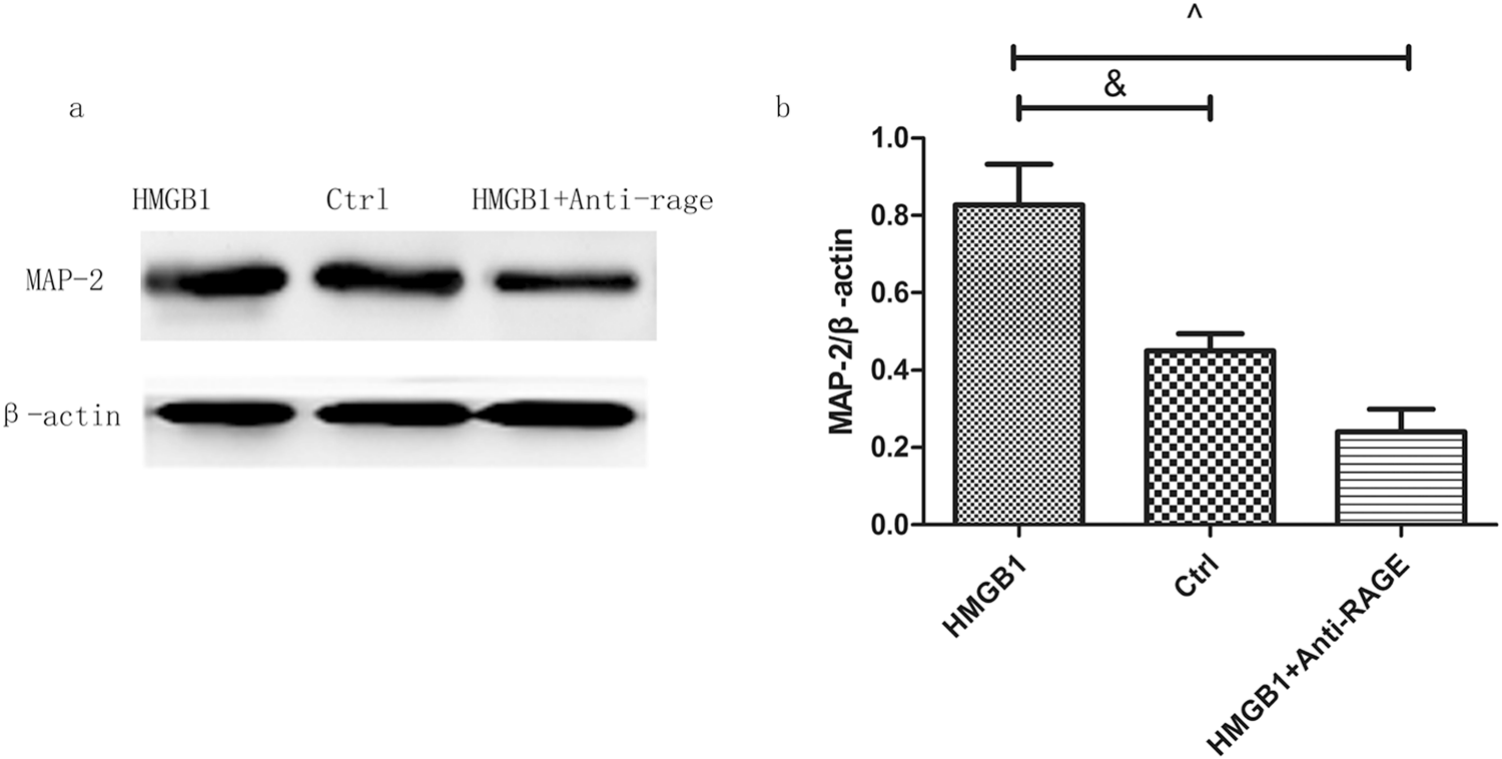
Recombinant HMGB1 protein promotes MAP-2 expressions in primary spinal neuron, but its effect can be blocked by RAGE antibody. (**a**) The expression of MAP-2 at 24 hours in the following groups: HMGB1; LPS and HMGB1 + anti-rage. (**b**) Quantitative analysis of MAP-2 expression in different groups. Values are means ± SD. ^&^P < 0.05, HMGB1 group vs ctrl group, ^^^P < 0.05, HMGB1 + anti-RAGE group vs HMGB1 group.

To explore the HMGB1/RAGE effect on MAP-2 expression in primary spinal neurons, via Western blot, we examined MAP-2 expression after being treated with recombinant protein HMGB1 (10 ng/ml) or anti-RAGE blocking antibody (10 µg/ml) for 24 hours, and we found that HMGB1 significantly increased MAP-2 expression (HMGB1 group vs ctrl group,82.74 ± 12.77%vs 45.05 ± 5.36%, p < 0.05; Fig. 7a,b) which was partly abolished by anti-RAGE treatment (anti-RAGE + HMGB1 group, 24.02 ± 7.15%, p < 0.05; Fig. 7a,b), indicating that HMGB1/RAGE signaling may promote MAP-2 mature neuron expression.

### RAGE blockade deteriorates motor neuron survival at the ventral horn of the injured spinal cord via Nissl staining

**Figure 8:**
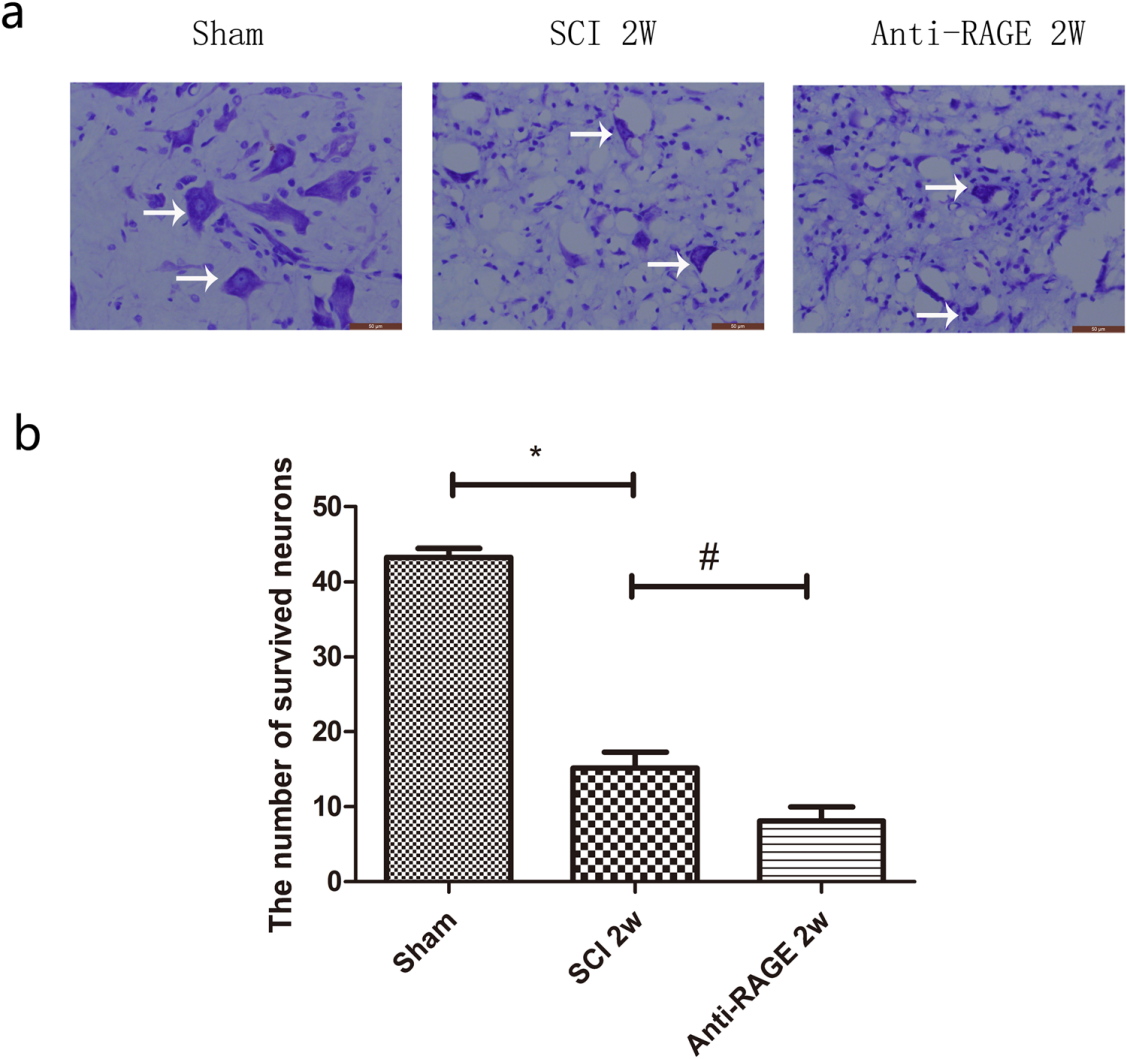
RAGE blockade damaged number of neuron survival at anterior horn of the spinal cord at 2 weeks via Nissl staining. (**a**) Figures with white arrows are neurons magnified by *400. Scar bar was 100 µm. (**b**) Quantitative analysis of the number of neuron survival at 2 weeks after SCI. Values are means ± SD. *P < 0.05, SCI group vs sham group, ^#^P < 0.05, anti-RAGE group vs SCI group.

To determine the effects of RAGE blockade in the injured spinal cord, we detected the motor neuron at the ventral horn of the injured spinal cord. In the sham group, a large number of Nissl-positive neurons with an extension cell body were expressed in the ventral horn, but their numbers were significantly reduced and most of them had an atrophic Nissl body. However, in the anti-RAGE group, Nissl-positive cells were seldom found, and their shape was withered (Fig. 8a). The surviving neurons of the ventral horn were detrimentally decreased after SCI (sham group vs SCI group, 43.28 ± 1.48 vs 8.58 ± 2.00, p < 0.05; Fig. 8b), but RAGE blockade showed a decreased number of surviving neurons at a higher magnification (anti-RAGE group vs SCI group, 6.45 ± 1.07 vs 8.58 ± 2.00; p < 0.05; Fig. 8b).

### RAGE blockade does not improve locomotor recovery function after SCI

**Figure 9:**
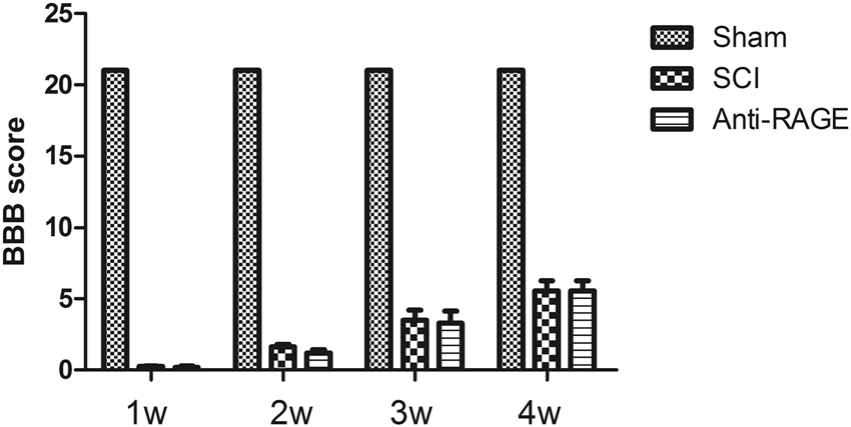
RAGE blockade showed no significant improvement in locomotor recovery until 4 weeks after SCI. Quantitative analysis of the BBB score showed no significant difference between anti-RAGE group and SCI group at 1, 2, 3 and 4 weeks. Values are means ± SD.

To examine the neurobehavioral function recovery, we examined the BBB score in the SCI rat, and we found that in the sham group, all the rats showed a score greater than 21 with no trouble in the movements of the hindlimb, while the score subsequently decreased after SCI with no movements in the hindlimb (sham group vs SCI group, 21 vs 0.2423 ± 0.064, p < 0.05; Fig. 9). RAGE blockade showed no significant difference compare with SCI group at the first week with a score of 0.2520 ± 0.1342 vs 0.2423 ± 0.064 (anti-RAGE group vs SCI group, p > 0.05; Fig. 9), at the second week with a score of 1.2291 ± 0.2786 vs 1.6594 ± 0.5732 (anti-RAGE group vs SCI group, p > 0.05; Fig. 9), at the third week with a score of 3.2941 ± 1.0457 vs 3.5135 ± 0.8576 (anti-RAGE group vs SCI group, p > 0.05; Fig. 9), at the fourth week with a score of 5.573 ± 0.8295 vs 5.572 ± 0.8429 (anti-RAGE group vs SCI group, p > 0.05; Fig. 9). RAGE inhibition does not improved locomotor function from the first week until the 4th week after SCI (Fig. 9).

## Discussion

RAGE is a transmembrane protein that is constitutively expressed during the embryonic stage, and levels decrease after growing up^34^. RAGE binding with various ligands sustained cellular activation in inflammation in acute spinal cord injury^27^. Interestingly, RAGE and its ligands were found to promote hippocampal neurogenesis in traumatic brain injury and to promote NSC proliferation and differentiation *in vitro* ^35^. HMGB1 and S100β bind with RAGE to mediate functions such as neurite outgrowth, migration, proliferation, and differentiation in the CNS^36, 37^. RAGE plays a detrimental role in the process of injury that depends on its concentration^38^. In our study, we found that RAGE blockade suppressed nestin-positive endogenous NSCs and their transformation into MAP-2-positive mature neurons. HMGB1 does not increase cytokines of TNF-a and IL-1β, but it promotes MAP-2-positive neuron expression through binding with RAGE in primary spinal neurons. RAGE deteriorates ventral neuron survival via Nissl staining and does not improve locomotor recovery after SCI. HMGB1/RAGE signaling may mediate NSC differentiation and promote neuronal recovery after SCI.

The NSCs can be activated, and their proliferation, migration and differentiation promote neurogenesis in CNS disease^13–16^. Studies showed that HMGB1 released from reactive astrocytes promotes NSC proliferation through binding with RAGE^26^. HMGB1/RAGE/NF-κB activation promotes subventricular zone region NSC proliferation and differentiation^35^. RAGE expression was co-expressed with NSC marker Sox2 in the hippocampus of adult mouse brains, and HMGB1/RAGE was involved in modulating NSC differentiation and adult neurogenesis^39^. In our study, we found that endogenous neuronal stem cells were expressed near the spinal central canal in the injured spinal cord, which modulates neurogenesis via transforming into neurons or other cell types. RAGE blockade treatment significantly suppressed the endogenous neuronal stem cell marker nestin, and RAGE was found to be activated 3 days after SCI (Fig. 1). In immunofluorescence double staining, the sham group seldom showed nestin expression and RAGE in the spinal cord. However, nestin-positive neural stem cells were highly co-expressed with RAGE, and RAGE blockade suppressed nestin/RAGE double-positive cell expression at 3 days after SCI, indicating that RAGE may have a role in regulating the number of NSCs (Fig. 2). SOX-2, a stem cell marker, has proven to regulate stem cell self-renewal and maintain its pluripotency^40^. In our study, we seldom found SOX-2-positive cells co-expressed with RAGE in the sham group. SOX-2/RAGE expression was significantly activated 2 weeks after SCI, and anti-RAGE treatment significantly reduced the percentage of SOX-2/RAGE double-positive cell expression (Fig. 3). We further observed nestin and MAP-2 expression by Western blot and IF double labeling. We found activated nestin and MAP-2 expression at 1 week after SCI, which was partly suppressed by RAGE blockade (Fig. 4). IF showed that anti-RAGE treatment also suppressed nestin expression that was activated after injury, but most nestin cells co-expressed with MAP-2 were partly suppressed by RAGE blockade at 1 week after SCI (Figs 4 and 5), suggesting that RAGE may regulate NSC differentiation into mature neurons. We determined MAP-2 expression after stimulating with HMGB1 and anti-RAGE antibody in primary spinal neuron culture; the results showed that HMGB1 does increase MAP-2 expression after 24 hours of treatment, but its effect was reversed after RAGE blockade (Fig. 7). These findings showed that HMGB1-RAGE may promote neural stem cells transformation into mature neurons. RAGE signaling may mediate the regeneration of the injured spinal cord by modulating neuronal stem cell differentiation.

RAGE is widely expressed in the neurons and astrocytes and plays an important role in axon regeneration and neurite outgrowth through binding with HMGB1^38, 41, 42^. HMGB1-RAGE interaction promotes neurite outgrowth and is highly co-expressed in the developing CNS^43^. HMGB1-RAGE mediates pro-inflammatory responses that contribute to secondary damage in the acute phase of SCI^31^. However, Dong, Y., *et al*. showed that HMGB1 does not mediate the inflammation in SCI but promotes neuronal regeneration^44^. RAGE and its ligands promote axon regeneration by regulating Schwann cells’ function in peripheral nerve injury^45^. With an *in vitro* study, we measured pro-inflammatory factors after stimulating with LPS or HMGB1 in primary spinal neuron culture. We tested pro-inflammatory cytokines of TNF-α and IL-1β in the medium, and we found that LPS exposure does increase the contents of TNF-α and IL-1β; however, HMGB1 does not increase the levels of TNF-α and IL-1β, implying that HMGB1 may not directly induce cytokines but that it may promote the effect of some inflammatory molecules^46^. However, RAGE blockade does decrease cytokines of TNF-α and IL-1β induced by LPS treatment (Fig. 6), suggesting that HMGB1/RAGE may not directly mediate pro-inflammatory factor release but may enhance the effects of pathogen-associated molecules.

However, the effect of RAGE on endogenous NSCs in SCI was still lacking study, and our study demonstrates that endogenous neural stem cells were activated 3 days after SCI and that RAGE blockade suppressed endogenous nestin-positive cells and attenuated its transformation into MAP-2-positive mature neurons. However, HMGB1 does not increase cytokines of pro-inflammatory factors but promotes MAP-2 expression in primary spinal neuron. HMGB1-RAGE signaling may somehow regulate NSC differentiation. RAGE antibody microinjection does not improve locomotor function and the BBB score exhibits no significance compared with the SCI vehicle group from 1 week to 4 weeks after SCI (Fig. 9). Though Guo,J.D. *et al*. report that RAGE knock down promotes neurobehavioral recovery in SCI^47^, we do not see the neuronal recovery by RAGE blockade; on the contrary, we discovered a reduced number of motor neurons at the ventral horn of the injured spinal cord after 2 weeks at the lesion site (Fig. 8). HMGB1/RAGE is beneficial for the neuronal survival at the ventral horn of the spinal cord by promoting NSC differentiation.

In conclusion, we demonstrate that there were nestin-positive endogenous stem cells and that they aid in neurogenesis near the central canal in SCI. HMGB1/RAGE play a critical role in modulating endogenous neural stem cell differentiation after SCI, while HMGB1 does not directly induce pro-inflammatory factors but promotes MAP-2-positive mature neurons in primary spinal neurons. The effects of HMGB1/RAGE on endogenous stem cells may promote neurogenesis and aid in functional recovery after SCI.

## Method and Materials

### Reagents and antibodies

Reagents used for cell culture were purchased from GIBCO (Invitrogen Corporation, Carlsbad, CA). Anti-RAGE-blocking antibody used in animals was purchased from RD System (Minneapolis, MN). LPS was purchased from Sigma–Aldrich (St. Louis, MO, USA); recombinant HMGB1 protein was purchased from Novus Biologicals (Colorado, USA). Anti-nestin antibody was purchased from Cell Signaling (Danvers, MA). Anti-RAGE antibody, anti-MAP-2 antibody, anti-SOX-2 antibody, and Tnf-a and IL-1β ELISA kits were purchased from Abcam (Cambridge, UK).

### Spinal cord injury model and experiment design

All the procedures were approved by the Animal Ethics Committee of Jinzhou Medical University. Adult male Sprague-Dawley rats 6–7 week of age, weighing 200–250 g were provided by Jinzhou Medical University. All experiment procedures were conducted in accordance with the guidelines of National Institutes of Health Guide for the Care and Use of Laboratory Animals (NIH Publication No. 85–23, revised 1996). Procedures were followed and contusion devices were used according to our previous papers^48^. Briefly, animals received a laminectomy at T9–10 segment after anesthetized by chloral hydrate (0.3 ml/kg). After the spinal cord was completely exposed, a weight-drop device was impacted in the T9–T10 segment, and anti-RAGE antibody or equal volume of 1 × PBS (phosphate-buffered saline) was injected immediately into the injured spinal cord. The wound was sutured and closed by layers. All rats received manual bladder expression three times a day until bladder function was reestablished.

### Primary spinal neuron culture

E14–15 Sprague-Dawley rats were collected by cesarean section. Procedures were described in our previous paper^49^. In brief, the spine tissues were digested in papain (Invitrogen, Carlsbad, CA) for 10 min at 37 °C, and 10% horse serum in minimum essential medium (MEM) was added to stop the digestion. After centrifugation at 1000 rpm for 5 min, cells were played on a disk coated with poly-D-lysine in a mixture of MEM, 10% horse serum, and 0.6% glucose supplemented with 1% penicillin/streptomycin. Cells at a density of 3 × 10^4^ cells/well in 100 mm culture dishes were plated for Western blot assessment. The cells’ medium was half changed every other day and was used for detection after being cultured for 1 week.

### Drug administration

Rats were automaticly divided into three groups (20 rats per group): the sham group, the SCI group, and the anti-RAGE group. In the sham group, a laminectomy operation was performed at the T9–10 segment; in the SCI group, contusion was made at the T9-10 segment and equal volume of 1 × PBS was microinjected into the lesion site immediately after injury. In the anti-RAGE group, after SCI at the T9-10 segment, anti-RAGE antibody (1 µg/ml; RD System) dissolved in 1.5 μl of 1 × PBS was injected immediately into the lesion site. Drugs were microinjected into the epicenter with a micro-injector fixed by stereotaxic apparatus with a rate of 0.5 µl/min.

In the primary spinal neuron culture, cells were treated with LPS (100ng/ml) for 12 hours; then, recombinant HMGB1 protein (10ng/ml) with or without anti-RAGE antibody (10ug/ml) was administered. 24 hours after incubation, we collected the medium supernatants or the proteins for detection.

### Nissl staining

The spinal cord tissues near the epicenter at a length of 1 cm were collected 2 weeks after SCI, and 10 µm thickness series sections were cut by the cryostat microtome. Procedures were performed as in our previous papers^50^. Briefly speaking, sections were soaked in 0.1% Cresyl violet Nissl staining solution, differentiated and dehydrated by 95% or 100% alcohol, and rinsed in xylene. The quantities of ventral horn neurons were calculated by randomly selecting four sections at the same site of each rat.

### Behavioral Test

The BBB locomotion rating scale was employed to evaluate the recovery condition of motor function after SCI at different time points (1w, 2w, 3w, and 4w) for each group; behavioral testing was analyzed as described previously^51^. Measures were performed by a double-blind independent examiner. Briefly speaking, BBB scores range from 0 points, (complete paralysis) to 21 points (normal locomotion). BBB scores at 1w, 2w, 3w, and 4w after SCI were detected and the average scores were calculated according to locomotor recovery after SCI.

### Western Blot

The injured site was extracted and dissolved in RIPA buffer for 30 min after machinery cutting and supersonic vibration. The protein concentration was qualified using the BCA kit. The proteins with a loading volume of 20 ul/well were separated by SDS–PAGE, and transferred to a PVDF membrane. Primary antibodies used were as follows: nestin (rabbit IgG, 1:1000; Cell Signaling, Danvers, MA); RAGE (rabbit IgG, 1:1000; Abcam, Cambridge, UK); or MAP-2 (rabbit IgG, 1:1000; Abcam, Cambridge, UK). After incubating with primary antibodies at 4 °C overnight and secondary antibodies at room temperature (RT) for 2 hours, and bands were observed by an ECL kit (Millipore) and quantified by ImageJ software.

### ELISA

Pro-inflammatory factors TNF-a and IL-1β in the medium were quantified using the TNF-a and IL-1β ELISA kit (Abcam, Cambridge, UK). Procedures were operated in accordance to the instructions of the manufacturer.

### Immunofluorescence Staining

The spinal tissues at the lesion site were extracted and stained with 4% paraformaldehyde and 30% sucrose. 10 µm thickness serial sections were collected and incubated with 0.1% Triton X-100 and 5% goat serum to penetrate the cell membrane for 2 hours and primary antibody at 4 °C overnight in a damp box. Primary antibodies were as follows: anti-nestin (mouse IgG; 1:400; Cell Signaling, Danvers, MA); anti-MAP-2 (rabbit IgG, 1:400; Abcam, Cambridge,UK); and anti-RAGE (rabbit IgG, 1:400; Abcam, Cambridge, UK). After washing with PBS for 3 × 10 min, samples were incubated with Alexa Fluor 488/568 FITC rabbit-anti-mouse secondary antibody (1:400) for 2 h at RT. Nuclei were counterstained by DAPI for 15 minutes. After three times rinsing with PBS, the glasses were covered with 50% glycerin. Pictures were screened on a Leica DMI4000B microscope and analyzed by Image J software.

### Statistical Analysis

Data were expressed as the mean ± SD and statistical diagram were formed by Graphpad Prism. SPSS (Version 17.0) were used to analyze the data and Student’s t-test (two groups) or one-way analysis of variance (ANOVA)(three groups and above) were performed to evaluate data. *P* values less than 0.05 were considered statistically significant.

### Data availability statement

The authors declare that the data was available.

## References

[1] Brambilla R (2005). Inhibition of astroglial nuclear factor kappaB reduces inflammation and improves functional recovery after spinal cord injury. The Journal of experimental medicine.

[2] Popovich PG, Longbrake EE (2008). Can the immune system be harnessed to repair the CNS?. Nature reviews. Neuroscience.

[3] Hausmann ON (2003). Post-traumatic inflammation following spinal cord injury. Spinal cord.

[4] Mosquera JA (2010). [Role of the receptor for advanced glycation end products (RAGE) in inflammation]. Investigacion clinica.

[5] Bierhaus A (2005). Understanding RAGE, the receptor for advanced glycation end products. Journal of molecular medicine (Berlin, Germany).

[6] Ott C (2014). Role of advanced glycation end products in cellular signaling. Redox biology.

[7] Xie J, Mendez JD, Mendez-Valenzuela V, Aguilar-Hernandez MM (2013). Cellular signalling of the receptor for advanced glycation end products (RAGE). Cellular signalling.

[8] Sorci G, Riuzzi F, Giambanco I, Donato R (2013). RAGE in tissue homeostasis, repair and regeneration. Biochimica et biophysica acta.

[9] Weiss S (1996). Multipotent CNS stem cells are present in the adult mammalian spinal cord and ventricular neuroaxis. The Journal of neuroscience: the official journal of the Society for Neuroscience.

[10] Clarke D, Frisen J (2001). Differentiation potential of adult stem cells. Current opinion in genetics & development.

[11] Clarke DL (2000). Generalized potential of adult neural stem cells. Science (New York, N.Y.).

[12] Johansson CB (1999). Identification of a neural stem cell in the adult mammalian central nervous system. Cell.

[13] Tattersfield AS (2004). Neurogenesis in the striatum of the quinolinic acid lesion model of Huntington’s disease. Neuroscience.

[14] Rice AC (2003). Proliferation and neuronal differentiation of mitotically active cells following traumatic brain injury. Experimental neurology.

[15] Jin K (2003). Directed migration of neuronal precursors into the ischemic cerebral cortex and striatum. Molecular and cellular neurosciences.

[16] Yagita Y (2001). Neurogenesis by progenitor cells in the ischemic adult rat hippocampus. Stroke.

[17] Schwartz PH (2003). Isolation and characterization of neural progenitor cells from post-mortem human cortex. Journal of neuroscience research.

[18] Yaworsky PJ, Kappen C (1999). Heterogeneity of neural progenitor cells revealed by enhancers in the nestin gene. Developmental biology.

[19] Fujiwara Y (2004). Intravenously injected neural progenitor cells of transgenic rats can migrate to the injured spinal cord and differentiate into neurons, astrocytes and oligodendrocytes. Neuroscience letters.

[20] Takahashi M, Arai Y, Kurosawa H, Sueyoshi N, Shirai S (2003). Ependymal cell reactions in spinal cord segments after compression injury in adult rat. Journal of neuropathology and experimental neurology.

[21] Englund U, Bjorklund A, Wictorin K (2002). Migration patterns and phenotypic differentiation of long-term expanded human neural progenitor cells after transplantation into the adult rat brain. Brain research. Developmental brain research.

[22] Roy NS (2000). *In vitro* neurogenesis by progenitor cells isolated from the adult human hippocampus. Nature medicine.

[23] Anthony TE, Klein C, Fishell G, Heintz N (2004). Radial glia serve as neuronal progenitors in all regions of the central nervous system. Neuron.

[24] Merkle FT, Tramontin AD, Garcia-Verdugo JM, Alvarez-Buylla A (2004). Radial glia give rise to adult neural stem cells in the subventricular zone. Proceedings of the National Academy of Sciences of the United States of America.

[25] Hasegawa K (2005). Embryonic radial glia bridge spinal cord lesions and promote functional recovery following spinal cord injury. Experimental neurology.

[26] Li M (2014). High-mobility group box 1 released from astrocytes promotes the proliferation of cultured neural stem/progenitor cells. International journal of molecular medicine.

[27] Song J, Lee WT, Park KA, Lee JE (2014). Receptor for advanced glycation end products (RAGE) and its ligands: focus on spinal cord injury. International journal of molecular sciences.

[28] Chen KB (2011). High-mobility group box-1 and its receptors contribute to proinflammatory response in the acute phase of spinal cord injury in rats. Spine.

[29] Rong LL (2004). RAGE modulates peripheral nerve regeneration via recruitment of both inflammatory and axonal outgrowth pathways. FASEB journal: official publication of the Federation of American Societies for Experimental Biology.

[30] Fages C, Nolo R, Huttunen HJ, Eskelinen E, Rauvala H (2000). Regulation of cell migration by amphoterin. Journal of cell science.

[31] Kawabata H (2010). High mobility group box 1 is upregulated after spinal cord injury and is associated with neuronal cell apoptosis. Spine.

[32] Gao HM (2011). HMGB1 acts on microglia Mac1 to mediate chronic neuroinflammation that drives progressive neurodegeneration. The Journal of neuroscience: the official journal of the Society for Neuroscience.

[33] Rauvala H, Pihlaskari R (1987). Isolation and some characteristics of an adhesive factor of brain that enhances neurite outgrowth in central neurons. The Journal of biological chemistry.

[34] Brett J (1993). Survey of the distribution of a newly characterized receptor for advanced glycation end products in tissues. The American journal of pathology.

[35] Meneghini V, Francese MT, Carraro L, Grilli M (2010). A novel role for the Receptor for Advanced Glycation End-products in neural progenitor cells derived from adult SubVentricular Zone. Molecular and cellular neurosciences.

[36] Saleh A (2013). Receptor for advanced glycation end-products (RAGE) activates divergent signaling pathways to augment neurite outgrowth of adult sensory neurons. Experimental neurology.

[37] Donato R (2001). S100: a multigenic family of calcium-modulated proteins of the EF-hand type with intracellular and extracellular functional roles. The international journal of biochemistry & cell biology.

[38] Huttunen HJ (2000). Coregulation of neurite outgrowth and cell survival by amphoterin and S100 proteins through receptor for advanced glycation end products (RAGE) activation. The Journal of biological chemistry.

[39] Meneghini V (2013). High-mobility group box-1 protein and beta-amyloid oligomers promote neuronal differentiation of adult hippocampal neural progenitors via receptor for advanced glycation end products/nuclear factor-kappaB axis: relevance for Alzheimer’s disease. The Journal of neuroscience: the official journal of the Society for Neuroscience.

[40] deCarvalho AC (2010). Gliosarcoma stem cells undergo glial and mesenchymal differentiation *in vivo*. Stem cells (Dayton, Ohio).

[41] Huttunen HJ, Fages C, Rauvala H (1999). Receptor for advanced glycation end products (RAGE)-mediated neurite outgrowth and activation of NF-kappaB require the cytoplasmic domain of the receptor but different downstream signaling pathways. The Journal of biological chemistry.

[42] Huttunen HJ, Rauvala H (2004). Amphoterin as an extracellular regulator of cell motility: from discovery to disease. Journal of internal medicine.

[43] Hori O (1995). The receptor for advanced glycation end products (RAGE) is a cellular binding site for amphoterin. Mediation of neurite outgrowth and co-expression of rage and amphoterin in the developing nervous system. The Journal of biological chemistry.

[44] Dong Y (2013). HMGB1 protein does not mediate the inflammatory response in spontaneous spinal cord regeneration: a hint for CNS regeneration. The Journal of biological chemistry.

[45] Perrone L, Peluso G, Melone MA (2008). RAGE recycles at the plasma membrane in S100B secretory vesicles and promotes Schwann cells morphological changes. Journal of cellular physiology.

[46] Tsan MF (2011). Heat shock proteins and high mobility group box 1 protein lack cytokine function. Journal of leukocyte biology.

[47] Guo JD (2014). Genetic ablation of receptor for advanced glycation end products promotes functional recovery in mouse model of spinal cord injury. Molecular and cellular biochemistry.

[48] Wang H (2015). VEGF inhibits the inflammation in spinal cord injury through activation of autophagy. Biochemical and biophysical research communications.

[49] Gao K (2016). Simvastatin inhibits neural cell apoptosis and promotes locomotor recovery via activation of Wnt/beta-catenin signaling pathway after spinal cord injury. Journal of neurochemistry.

[50] Li D (2016). MP Resulting in Autophagic Cell Death of Microglia through Zinc Changes against Spinal Cord Injury. BioMed research international.

[51] Gao K (2015). Neuroprotective Effect of Simvastatin via Inducing the Autophagy on Spinal Cord Injury in the Rat Model. BioMed research international.

